# Supplementary material for: Clinical Features of COVID-19 Vaccine-Associated Autoimmune Hepatitis: A Systematic Review
Source: Diseases. 2023 May 30;11(2):80. doi: 10.3390/diseases11020080 (PMC10297001; doi:10.3390/diseases11020080)
Supplement: Supplementary file 1 [file diseases-11-00080-s001.zip › diseases-2346385-supplementary.pdf]

**First author**	**Country/Years**	**Gender/Age**	**Personal history**	**routine medication**	**Type of COVID-19 vaccine**	**Timing of Symptom Onset**	**Symptoms**	**Hepatotropic viruses**	**Peak laboratory values**	**Autoantibodies Positive**	**Liver biopsy**	**Treatment**	**Outcome**
**Garrido [1]**	Portugal/2021	F/65	Polycythemia vera	Acetylsalicylic acid;<br>Sertraline;<br>Esomeprazole;<br>Pegylated interferon	Moderna	Day 14 after 1st does	Jaundice;<br>Choluria;<br>Abdominal pain;	HIV;HAV;HBV;HCV;<br>HEV;CMV;EBV;HSV	ALT:1092 U/L<br>AST:1056 U/L<br>ALP:24 U/L<br>TBL:1.14 mg/dl<br>GGT:329 U/L<br>IgG:2000 mg/dl	ANA	Intense plasma cells infiltrate in the poral;<br>Severe interface hepatitis;<br>Multiple confluent foci of lobular necrosis;	Prednisolone	Improved
**Avci[2]**	Turkey/2021	F/61	Hashimoto's thyroiditis;<br>Hypertension;<br>Infected COVID-19	Valsartan;<br>Levothyroxine	Pfizer	Day 14 after 1st does	Jaundice;<br>Malaise;<br>Fatigue;<br>Anorexia;<br>Nausea;	HAV;HBV;HCV;<br>CMV;EBV	ALT:455<br>AST:913<br>TBL:11.8<br>GGT:292<br>IgG:4260	ANA<br>ASMA	Intense lymphocyte infiltrate in the poral and periportal;<br>Periseptal interface hepatitis;<br>Spotty necrosis;<br>Fibrosis	Prednisolone;<br>Azathioprine;	Improved
**Bril [3]**	USA/2021	F/25	Postpartum;<br>gestational hypertension	Labetalol	Pfizer	Day 7 after 1st does	Pruritus;<br>Choluria;<br>Jaundice	HIV;HAV;HBV;HCV;<br>CMV;EBV;HSV	ALT:2001<br>AST:754<br>ALP:170<br>TBL:4.8<br>IgG:1081	ANA;<br>ds-DNA.	Intense lymphoplasmacytic, eosinophils and plasma cells infiltrate in the portal;<br>Scattered necrotic hepatocytes	Prednisone	Improved
**Ghielmetti. [4]**	Switzerland/2021	M/63	Type 2 diabetes;<br>ischemic heart disease	Metformin;<br>Acetylsalicylic acid;<br>Rosuvastatin	Moderna	Day 7 after 1st does	Jaundice;<br>Fatigue;<br>Anorexia;	anti-HAV IgM;<br>HBV;HCV;HEV;	ALT:1038<br>AST:1127<br>ALP:192<br>TBL:2.32<br>GGT:536<br>IgG:19.96	ANA;<br>ASMA	Intense lymphoplasmacytic and plasma cellular<br>infiltrate with scattered eosinophils;<br>Interface hepatitis;<br>Centrilobular necrosis;	Prednisone	Improved
**Rocco[5]**	Italy/2021	F/80	Hashimoto's thyroiditis;<br>acute glomerulonephritis	Levothyroxine;<br>Corticosteroids;<br>Pravastatin;<br>Aspirin	Pfizer	Day 7 after 1st does	Choluria;<br>Jaundice	HIV;HAV;HBV;HCV;<br>;CMV;EBV;HSV	ALT:1186<br>AST:1401<br>ALP:243<br>TBL:10.5<br>GGT:524<br>IgG:3500	ANA;	Lymphoplasmacytic infiltrate;<br>Multiple confluent foci of lobular necrosis and councilman bodies;	Prednisone	Improved
**Tan[6]**	Singapore/2021	F/56	NA	Rosuvastatin	Pfizer	Day 1 after 1st does	Malaise;<br>Fever;<br>Fatigue;<br>Anorexia<br>Jaundice	HAV;HBV;HCV;<br>HEV;CMV;EBV;HSV	ALT:1701<br>AST:1124<br>ALP:298<br>TBL:1.15<br>IgG:3260	ANA;<br>ASMA;	Intense plasma cells, lymphocytes and few eosinophils aggregation in portal tract;<br>interface hepatitis;<br>fibrosis;	Budesonide	Improved
**Glayton [7]**	Australia/2021	M/36	Hypertension;<br>Laser eye surgery;	Olmesartan;<br>Acetaminophen;<br>Ibuprofen;	AstraZeneca	Day 26 after 1st does	Pruritus	HIV;HAV;HBV;HCV;<br>HEV;CMV;EBV;HSV	ALT:2550<br>AST:633<br>ALP:118<br>TBL:45<br>GGT:136<br>IgG:1280	ANA;	Intense predominantly lymphocytic in the portal tract;<br>severe interface hepatitis;<br>fibrosis;	Prednisolone	Improved
**Zin[8]**	UK/2021	M/47	NA	NA	Moderna	Day 3 after 1st does;<br>within day 7 after 2nd does	Malaise;<br>Jaundice;<br>Abdominal pain;	HAV;HBV;HCV; HEV<br>;CMV;EBV;	ALT:1048<br>ALP:229<br>TBL:41<br>IgG:2510	ANA;	Lymphoplasmatic inflammation including eosinophils, ballooned hepatocytes, multinucleated giant cells, and emperipolesis;<br>marked interface hepatitis;<br>minimal fibrosis;	Prednisolone	Improved
**Lodato [9]**	Italy/2021	F/43	Hyperlipidemia;	Ginkgo-biloba	Pfizer	Day 15 after 1st does;<br>day 2 after 2nd does	Malaise;<br>Jaundice;<br>Abdominal pain;	HIV;HAV;HBV;HCV;<br>HEV;CMV;EBV;	ALT:171<br>AST:132<br>TBL:29.15 mg/dl	Negative	Moderate lymphocytes infiltrate in the portal; interface hepatitis; spotty necrosis; focal moderate steatosis; no fribrosis	N-acetylcysteine;<br>Methylprednisolone	Improved

**Ehelfi [10]**	Germany/2021	F/36	Primary sclerosing cholangitis;<br>Ulcerative colitis	Ursodeoxycholic acid;<br>Endoscopic biliary intervention;	Moderna	Day 11 after 1st does	Jaundice;<br>Pruritus;<br>Fatigue;	HAV;HBV;HCV;HEV;<br>CMV;EBV;	ALT: 588<br>AST: 581<br>ALP: 1077<br>TBL: elvated<br>GGT: 757<br>IgG: 2475	ANA;<br>ds-DNA	Intense lymphoplasma cellular, plasma cells, few eosinophils infiltrate in the portal small bile ducts; interface hepatitis;scatter rosette formation and apoptotic hepatocytes;	Prednisone	Improved
**Mahalingham[11]**	UK/2021	F/32	Orthotopic liver transplantation for autoimmune hepatitis;<br>Hypertension;	Tacrolimus;<br>Azathioprine;<br>Prednisolone;<br>Aspirin;<br>Vitamin D;<br>Atenolol;	Pfizer	Day 26 after 2nd does	NA	HAV;HBV;HCV;HDV;<br>HEV;CMV;EBV;	NA	Soluble liver antigen;<br>Liver pancreas antibody positive	Intense lymphoplasmacytic, plasma cells, rare eosinophils infiltrate in the poral; interface activity with focal rosetting and emperipolesis; No documented fibrosis;	Methylprednisolone; Prednisolone;<br>Azathioprine	Improved
**Fimiano [12]**	Italy/2021	F/63	postmenopausal hypothyroidism;<br>Familial autoimmune conditions;	NA	Pfizer	Day 53 after 1st does	Abdominal pain;<br>Anorexia;<br>Choluria;<br>Jaundice	HIV;HAV;HBV;HCV;<br>HEV;CMV;EBV;HSV;HHV;	ALT:1778<br>AST:1625<br>ALP:273<br>TBL:18.6<br>GGT:419<br>IgG:1760	ANA;	Intense plasma cells infiltrate in the portal/periportal and lobular; interface activity, focal confluent necrosis, scattered apoptotic bodies;	methylprednisolone;<br>Azathioprine	Improved
**Erard [13]**	France/2021	F/80	NA	NA	Pfizer	Day 10 after 1st does	Fatigue;<br>Pruritus;<br>Jaundice;	HIV;HAV;HBV;HCV;HEV;<br>CMV;EBV;HSV	ALT:541<br>AST:583<br>TBL:0.88<br>IgG:2090	ANA;	Intense lymphoplasmacytic infiltrate in the lobular and portal; interface hepatitis; hepatocyte necrosis;	Steroids	Improved
		F/73	NA	NA	Moderna	Day 21 after 1st does	Fatigue;<br>Pruritus;<br>Jaundice;	HIV;HAV;HBV;HCV;HEV;<br>CMV;EBV;HSV	ALT:1027<br>AST:1163<br>TBL:3.78<br>IgG:1800	ANA;	Intense lymphoplasmacytic infiltrate in the lobular and portal; interface hepatitis; hepatocyte necrosis;	Steroids	Improved
		F/68	NA	NA	AstraZeneca	Day 20 after 1st does	Fatigue;<br>Pruritus;<br>Jaundice;	HIV;HAV;HBV;HCV;HEV;<br>CMV;EBV;HSV	ALT:2029<br>AST:2314<br>TBL:8.5<br>IgG:1850	ANA;	Intense lymphoplasmacytic infiltrate in the lobular and portal; interface hepatitis; hepatocyte necrosis;	Steroids	Died
**Kang [14]**	Korea/2021	F/27	None	None	BioNTech	Day 7 after 2nd does	Jaundice;	HIV;HAV;HBV;HCV;HEV;<br>CMV;EBV;HSV	ALT:1478<br>AST:1004<br>ALP:182<br>TBL:8.6<br>IgG:1641	ANA;	Intense lymphocytes, clusters of plasma cells and few eosinophils infiltrate in the portal tract and lobules; mild cholestasis with hepatocytic rosettes; interface hepatitis; mild cholestasis with hepatocytic rosettes; moderate periportal fibrosis;	Prednisolone	Improved
**Shahrani[15]**	Malaysia/2022	F/59	Dyslipidemia	NA	AstraZeneca	Day 12 after 2nd does	Jaundice	HAV;HBV;HCV;HEV	ALT:1178<br>AST:962<br>IgG:1740	NA	Lympho-plasma-cells infiltrate in the protral;	Prednisolone	Improved
		F/63	Ulcerative colitis;<br>Primary sclerosing cholangitis ;	NA	AstraZeneca	Day 14 after 1st does	Jaundice	HAV;HBV;HCV;HEV	ALT:354<br>AST:505<br>IgG:2030	NA	Presence of interface and lobular hepatitis; Bridging fibrosis;	Prednisolone	Died

		F/72	NA	NA	Pfizer	Day 10 after 1st does	Jaundice	HAV;HBV;HCV;HEV	ALT:2280<br>AST:254<br>IgG:1940	NA	Intense of lymphocytes and plasma cells in the protal;<br>hepatocyte necrosis;	Prednisolone	Improved
**Bjornsson [16]**	Italy/2022	F/52	lung adenocarcinoma;<br>HBV with normal liver function test	Carboplatin;<br>Pemetrexed;<br>Pembrolizumab	Pfizer	Day 10 after 1st does	Diarrhea	HAV;HCV;HDV;HEV	ALT:229<br>AST:147<br>ALP:161<br>TBL:1.98<br>GGT:139<br>IgG:1400	ANA<br>ASMA	Portal inflammation;	Prednisone	Improved
**Goulas [17]**	Greece/2022	F/52	None	None	Moderna	Day 14 after 1st does	Malaise;<br>Jaundice	HAV;HBV;HCV;HEV;CMV;EBV;	ALT:936<br>AST:350<br>ALP:169<br>TBL:9.06<br>GGT:810<br>IgG:2396	ANA;<br>ASMA	Intense lymphocytes, plasmatocytes, histiocytes, few eosinophiles and neutrophiles infiltrate in the portal;<br>mild periportal hepatitis; apoptotic bodies;hepatocytes with feathery degeneration of the cytoplasm and rosette formation	Prednisolone<br>Azathioprine	Improved
**Pinazo [18]**	Spain/2022	F/77	None	Bromazepam;<br>Losartan;<br>Omeprazole	Pfizer	Day 2 after 2nd does	NA	Undocumented	ALT:552<br>AST:474<br>ALP:159<br>TBL:3.1<br>IgG:Normal	ANA;<br>AMAs	Compatible with AIH	Prednisone;<br>Azathioprine;<br>Budesonide	Died
		M/23	None	None	Moderna	Day 10 after 2nd does	NA	Undocumented	ALT:587<br>AST:702<br>ALP:202<br>TBL:2.3<br>IgG:1647	NA	Compatible with AIH	Prednisone	Improved
**Bjornsson [19]**	Singapore/2022	M/34	Drank socially	None	Moderna	Day 16 after 2nd does	Pruritus;<br>Fever;<br>Jaundice	HAV;HBV;HCV;<br>HEV;CMV;EBV;	ALT:223<br>AST:111<br>ALP:149<br>TBL:1.49<br>GGT:98<br>IgG:1460	Negative	Mild portal inflammation with scattered eosinophils, cholestasis, scattered eosinophils;	Ursodeoxycholic acid;	Improved
**Londono[20]**	Spain/2022	F/41	Premature ovarian failure	Substitutive hormonal therapy	Moderna	Day 7 after 2nd does	Abdominal pain;<br>Nausea<br>Choluria;<br>Jaundice	"HIV;HAV;HBV;HCV;HEV;CMV;EBV;HSV"	ALT:1312<br>AST:993<br>ALP:190<br>TBL:2.3<br>GGT:209<br>IgG:2080	ANA;<br>ASMA;<br>anti-SLA;<br>anti-LC1	Intense lymphocytes and plasma cells infiltrate in the portal tracts; severe interface hepatitis; apoptotic bodies; perisinusoidal fibrosis;	Prednisone	Improved
**Mcshen[21]**	Ireland/2021	F/71	left total hip replacement;<br>osteoarthritis of the knees;<br>HAV IgG(+)	None	Moderna	Day 10 after 1st does	Jaundice	"HIV;HBV;HCV;<br>HEV;CMV;EBV;HSV"	ALT:1143<br>AST:1469<br>ALP:237<br>TBL:3.76<br>IgG:2177	ANA;<br>ASMA	Intense plasma cells, lymphocytes, eosinophils, neutrophils infiltrate in the portal; interface hepatitis; rosettes formed; bridging necrosis;	Prednisolone	Improved
**Rela [22]**	India/2021	F38	Hypothyroidism	Levothyroxine	AstraZeneca	Day 20 after 1st does	Fever; Fatigue;<br>Jaundice	HAV;HBV;HCV; HEV	ALT:1025<br>AST:1101<br>TBL:14.9<br>IgG:1650	ANA	Intense lymphocytes, plasma cells and rare eosinophils infiltrate in the portal; multiacinar hepatic necrosis; diffuse portal/periportal neocholangiolar proliferation;	Prednisolone	Improved

		M/62	Jaundice	None	AstraZeneca	Day 16 after 1st does	Fever; Anorexia; Jaundice	HAV; HBV; HEV; HCV;	ALT:1094 AST:1361 TBL:25.6	Negative	Moderate lymphocytes; plasma cells, eosinophils and polymorphs infiltrate in the portal; porto-central bridging necrosis; mild fibrosis;	Prednisolone	Died
**Palla [23]**	Greece/ 2022	F/40	Sarcoidosis	None	Pfizer	NA	NA	HIV;HAV;HBV;HCV;HEV; CMV;EBV;HSV	ALT:4X upper limit of normal AST:4X upper limit of normal IgG:2400	ANA;	Intense lymphocytes; plasma cells infiltrate in the portal and lobular interface hepatitis; Portal and periportal fibrosis;	Prednisolone	Improved
**Vuille [24]**	Switzerl and/202 1	F/76	COVID-19 infected; Hashimoto thyroiditis; urothelial carcinoma;	Levothyroxine, Midodrine; Zolpidem	Moderna	Day 3 after 1st does	NA	HAV;HBV;HCV; HEV;CMV;	ALT:579 AST:811 ALP:124 TBL:0.74 GGT:361 IgG:3940	ANA ASMA ANCA	Intense plasma cells infiltrates in the protral; interface hepatitis; apoptotic bodies; pseudorosettes	Prednisolone; Azathioprine	Improved
**Shroff [25]**	USA/20 21	M/46	Nonalcoholic fatty liver disease; Drug-induced liver injury	Amoxicillin	Pfizer	Day 10 after 1st does	NA	Viral serologies negative	ALT:594 ALP:197 TBL:3.9	ASMA	Mixed infiltrate in the portal; Mild ductular proliferation; portal and peri-portal fibrosis;	Endoscopic biliary dilation	Improved
		F/61	None	Nitrofurantoin	Pfizer	Day 34 after 1st does	NA	Viral serologies negative	ALT:2331 ALP:160 TBL:3.7	ASMA	Lymphocytes and plasma cells in the portal and lobular;	Prednisone	Improved
		F/42	None	None	Pfizer	Day 42 after 1st does	NA	EBV(+)	ALT:1735 ALP:287 TBL:1.5	ANA	Intense mixed infiltrate in the portal;Neutrophilic peri-cholangitis;	Prednisone	Improved
		F/33	AIH (treated) Compensated cirrhosis	None	Pfizer	Day 28 after 1st does	NA	None documented	ALT:173 ALP:46 TBL:2.1	NA	Intense plasma cells in the portal and lobular; interface hepatitis; cirrhosis;	Prednisone	Improved
		M/68	Diagnosis of solitary hepatocellular carcinoma	None	Pfizer	Day 19 after 1st does	NA	Viral serologies negative	ALT:245 ALP:55 TBL:0.9	NA	Intense plasma cells in the portal and lobular;interface hepatitis; cirrhosis;	Prednisone	Improved
		F/66	AIH (treated)	Received shingles vaccine	Moderna	Day 5 after 1st does	NA	Viral serologies negative	ALT:1199 ALP:352 TBL:5.9	NA	Intense plasma cells in the portal and lobular;interface hepatitis; central perivenulitis;	Prednisone	Improved

		F/68	Urinary tract infection	Ceftriaxone	Moderna	Day 15 after 1st does	NA	Viral serologies negative	ALT:2367<br>ALP:176<br>TBL:25	Negative	Intense inflammtion in the portal and lobular; interface hepatitis; severe bile; ductular recction	steroids;<br>N-acetylcysteine	Improved
		F/59	None	Tylenol	Moderna	Day 31 after 1st does	NA	EBV(+);<br>Viral serologies negative	ALT:869<br>ALP:367<br>TBL:14.7	ANA	Intense Lymphocytes in the portal and lobular; Ductular reaction;	Methylprednisolone	Improved
		M/59	None	Tylenol	Moderna	Day 46 after 1st does	NA	Viral serologies negative	ALT:2664<br>ALP:2252<br>TBL:22.3	ANA;<br>ASMA	Mild Lymphocytes in the portal;<br>bile duct injury;	None	Improved

Abbreviations: M, male; F, female; AIH, autoimmune hepatitis; ALT, alanine aminotransferase; AST, aspartate aminotransferase; ALP, alkaline phosphatase; TBL, total bilirubin; GGT, gamma glutamyl transferase; IgG, immunoglobulin G; ANA, anti-nuclear antibodies; ASMA, anti-smooth muscle antibodies; ds-DNA; anti-double stranded DNA; anti-SLA, antibodies against soluble liver antigen; anti-LC1, anti-liver cytosol type 1.

1. Garrido, I., et al., *Autoimmune hepatitis after COVID-19 vaccine - more than a coincidence.* J Autoimmun, 2021. **125**: p. 102741.
2. Avci, E. and F. Abasiyanik, *Autoimmune hepatitis after SARS-CoV-2 vaccine: New-onset or flare-up?* J Autoimmun, 2021. **125**: p. 102745.
3. Bril, F., et al., *Autoimmune hepatitis developing after coronavirus disease 2019 (COVID-19) vaccine: Causality or casualty?* J Hepatol, 2021. **75**(1): p. 222-224.
4. Ghielmetti, M., et al., *Acute autoimmune-like hepatitis with atypical anti-mitochondrial antibody after mRNA COVID-19 vaccination: A novel clinical entity?* J Autoimmun, 2021. **123**: p. 102706.
5. Rocco, A., et al., *Autoimmune hepatitis following SARS-CoV-2 vaccine: May not be a casuality.* J Hepatol, 2021. **75**(3): p. 728-729.
6. Tan, C.K., et al., *Autoimmune hepatitis following COVID-19 vaccination: True causality or mere association?* J Hepatol, 2021. **75**(5): p. 1250-1252.
7. Clayton-Chubb, D., et al., *Autoimmune hepatitis developing after the ChAdOx1 nCoV-19 (Oxford-AstraZeneca) vaccine.* J Hepatol, 2021. **75**(5): p. 1249-1250.
8. Zin Tun, G.S., et al., *Immune-mediated hepatitis with the Moderna vaccine, no longer a coincidence but confirmed.* J Hepatol, 2022. **76**(3): p. 747-749.
9. Lodato, F., et al., *An unusual case of acute cholestatic hepatitis after m-RNABNT162b2 (Comirnaty) SARS-CoV-2 vaccine: Coincidence, autoimmunity or drug-related liver injury.* Journal of Hepatology, 2021. **75**(5): p. 1254-1256.
10. Ghelfi, J., et al., *Successful management of refractory ascites in non-TIPSable patients using percutaneous thoracic duct stenting.* J Hepatol, 2022. **76**(1): p. 216-218.
11. Mahalingham, A., A. Duckworth, and W.J.H. Griffiths, *First report of post-transplant autoimmune hepatitis recurrence following SARS-CoV-2 mRNA vaccination.* Transpl Immunol, 2022. **72**: p. 101600.
12. Fimiano, F., et al., *Autoimmune hepatitis or drug-induced autoimmune hepatitis following Covid-19 vaccination?* Liver Int, 2022. **42**(5): p. 1204-1205.
13. Erard, D., et al., *Autoimmune hepatitis developing after COVID 19 vaccine: Presumed guilty?* Clin Res Hepatol Gastroenterol, 2022. **46**(3): p. 101841.
14. Kang, S.H., et al., *Autoimmune Hepatitis Following Vaccination for SARS-CoV-2 in Korea: Coincidence or Autoimmunity?* J Korean Med Sci, 2022. **37**(15): p. e116.
15. Shahrani, S., et al., *Autoimmune hepatitis (AIH) following coronavirus (COVID-19) vaccine-No longer exclusive to mRNA vaccine?* Liver Int, 2022. **42**(10): p. 2344-2345.
16. Lasagna, A., *Development of hepatitis triggered by SARS-CoV-2 vaccination in patient with cancer during immunotherapy: a case report.* , 2022.
17. Goulas, A., et al., *A typical autoimmune hepatitis (AIH) case following Covid-19 mRNA vaccination. More than a coincidence?* Liver Int, 2022. **42**(1): p. 254-255.
18. Pinazo-Bandera, J.M., et al., *Acute hepatitis with autoimmune features after COVID-19 vaccine: coincidence or vaccine-induced phenomenon?* Gastroenterol Rep (Oxf), 2022. **10**: p. goac014.
19. Bjornsson, H.K. and E.S. Bjornsson, *Reply to: "Can azathioprine prevent infliximab-induced liver injury?".* J Hepatol, 2022. **77**(2): p. 555-556.
20. Londono, M.C., J. Gratacos-Gines, and J. Saez-Penataro, *Another case of autoimmune hepatitis after SARS-CoV-2 vaccination - still casualty?* J Hepatol, 2021. **75**(5): p. 1248-1249.
21. McShane, C., et al., *The mRNA COVID-19 vaccine - A rare trigger of autoimmune hepatitis?* J Hepatol, 2021. **75**(5): p. 1252-1254.
22. Rela, M., et al., *Auto-immune hepatitis following COVID vaccination.* J Autoimmun, 2021. **123**: p. 102688.
23. Palla, P., et al., *Letter to the editor: Autoimmune hepatitis after COVID-19 vaccination: A rare adverse effect?* Hepatology, 2022. **75**(2): p. 489-490.
24. Vuille-Lessard, E., et al., *Autoimmune hepatitis triggered by SARS-CoV-2 vaccination.* J Autoimmun, 2021. **123**: p. 102710.
25. Shroff, H., et al., *Liver injury following SARS-CoV-2 vaccination: A multicenter case series.* J Hepatol, 2022. **76**(1): p. 211-214.
